# Characterization of the Adherence of *Clostridium difficile* Spores: The Integrity of the Outermost Layer Affects Adherence Properties of Spores of the Epidemic Strain R20291 to Components of the Intestinal Mucosa

**DOI:** 10.3389/fcimb.2016.00099

**Published:** 2016-09-22

**Authors:** Paola Mora-Uribe, Camila Miranda-Cárdenas, Pablo Castro-Córdova, Fernando Gil, Iván Calderón, Juan A. Fuentes, Paula I. Rodas, Saeed Banawas, Mahfuzur R. Sarker, Daniel Paredes-Sabja

**Affiliations:** ^1^Microbiota-Host Interactions and Clostridia Research Group, Departamento de Ciencias Biológicas, Facultad de Ciencias Biológicas, Universidad Andres BelloSantiago, Chile; ^2^Center for Bioinformatics and Integrative Biology, Facultad de Ciencias Biológicas, Universidad Andres BelloSantiago, Chile; ^3^Laboratorio de Genética y Patogénesis Bacteriana, Departamento de Ciencias Biológicas, Facultad de Ciencias Biológicas, Universidad Andres BelloSantiago, Chile; ^4^Facultad de Medicina, Center for Integrative Medicine and Innovative Sciences, Universidad Andres BelloSantiago, Chile; ^5^Department of Biomedical Sciences, Oregon State UniversityCorvallis, OR, USA; ^6^Medical Laboratories Department, College of Science Al-Zulfi, Majmaah UniversityAl Majma′ah, Saudi Arabia

**Keywords:** *C. difficile* spores, exosporium, spore adherence, CdeC, BclA

## Abstract

*Clostridium difficile* is the causative agent of the most frequently reported nosocomial diarrhea worldwide. The high incidence of recurrent infection is the main clinical challenge of *C. difficile* infections (CDI). Formation of *C. difficile* spores of the epidemic strain R20291 has been shown to be essential for recurrent infection and transmission of the disease in a mouse model. However, the underlying mechanisms of how these spores persist in the colonic environment remains unclear. In this work, we characterized the adherence properties of epidemic R20291 spores to components of the intestinal mucosa, and we assessed the role of the exosporium integrity in the adherence properties by using *cdeC* mutant spores with a defective exosporium layer. Our results showed that spores and vegetative cells of the epidemic R20291 strain adhered at high levels to monolayers of Caco-2 cells and mucin. Transmission electron micrographs of Caco-2 cells demonstrated that the hair-like projections on the surface of R20291 spores are in close proximity with the plasma membrane and microvilli of undifferentiated and differentiated monolayers of Caco-2 cells. Competitive-binding assay in differentiated Caco-2 cells suggests that spore-adherence is mediated by specific binding sites. By using spores of a *cdeC* mutant we demonstrated that the integrity of the exosporium layer determines the affinity of adherence of *C. difficile* spores to Caco-2 cells and mucin. Binding of fibronectin and vitronectin to the spore surface was concentration-dependent, and depending on the concentration, spore-adherence to Caco-2 cells was enhanced. In the presence of an aberrantly-assembled exosporium (*cdeC* spores), binding of fibronectin, but not vitronectin, was increased. Notably, independent of the exosporium integrity, only a fraction of the spores had fibronectin and vitronectin molecules binding to their surface. Collectively, these results demonstrate that the integrity of the exosporium layer of strain R20291 contributes to selective spore adherence to components of the intestinal mucosa.

## Introduction

The Gram-positive, anaerobic, spore-forming bacterium, *Clostridium difficile*, is the leading cause of health care-associated infections world-wide (Evans and Safdar, 2015). The clinical manifestation of *C. difficile* infections (CDI) vary from mild to severe diarrhea, which can lead to fulminant colitis, toxic megacolon, bowel perforation, sepsis and death (Rupnik et al., 2009). The mortality rates of CDI reach 5% of total cases, but during outbreaks it may reach up to 20% (Pepin et al., 2005). Current antibiotic therapies, although effectively eradicate the infection, lead to CDI recurrence after a first episode in ~20–30% of the patients (Evans and Safdar, 2015), which is one of the main current clinical challenges in CDI treatment (Barra-Carrasco and Paredes-Sabja, 2014). CDI is primarily a toxin-mediated disease, however, during the infectious cycle, *C. difficile* begins to produce metabolically dormant spores through the initiation of the sporulation process (Deakin et al., 2012; Paredes-Sabja et al., 2014) which has been linked to be essential for CDI recurrence (Deakin et al., 2012).

The mechanism(s) involved in the persistence of *C. difficile* spores in the host are still unclear, but it is thought that the outermost exosporium-like layer plays an important role in spore persistence. Recent studies have provided evidence of several biological aspects of this outermost layer. A recent proteomic study demonstrated that this outermost exosporium is a proteinaceous layer (Diaz-Gonzalez et al., 2015), that can be removed by enzymatic or mechanical treatments and contributes to the hydrophobicity of the spore surface (Escobar-Cortes et al., 2013). The exosporium layer of *C. difficile* spores has several differences and similarities with previously reported outermost surfaces (i.e., the crust layer of *B. subtilis* spores and the exosporium layer of spores of the *B. cereus* group). First, unlike the exosporium layer of the *B. cereus* group, which has a hair-like nap separated from the spore coat by an interspace gap, the *C. difficile* exosporium-like layer surrounds the spore coat surface in a similar manner as the outer crust of *B. subtilis* spores (Stewart, 2015). Second, the *C. difficile* exosporium layer, similarly as in spores of the *B. cereus* group, has hair-like projections (Henriques and Moran, 2007; Paredes-Sabja et al., 2014; Stewart, 2015; Pizarro-Guajardo et al., 2016). In the *B. cereus* group, these hair-like extensions are formed by the collagen-like BclA glycoproteins (Henriques and Moran, 2007; Stewart, 2015); orthologs of the BclA family are highly conserved in the *C. difficile* sequenced genomes and have been located exclusively in the exosporium proteome (Diaz-Gonzalez et al., 2015). Third, proteins of this outermost exosporium layer of *C. difficile* spores have been shown to be relevant for spore-host interactions (Phetcharaburanin et al., 2014), similarly as observed in the spores of the *B. cereus* group (Stewart, 2015). Fourth, the composition of this outermost exosporium layer in *C. difficile*, although has orthologs of the BclA family of proteins, it lacks orthologs of structural proteins, indicating that novel uncharacterized proteins are structural determinants of this layer (Diaz-Gonzalez et al., 2015). Fifth, the assembly of this exosporium-like layer requires the presence of the cysteine-rich protein CdeC (Barra-Carrasco et al., 2013). *C. difficile cdeC* spores have an aberrantly-assembled exosporium layer that lacks electron dense material and the hair-like projections (Barra-Carrasco et al., 2013), yet the biochemical properties of this protein and the mechanisms underlying its role in exosporium assembly remain unclear.

Few studies have addressed how *C. difficile* spores interact with components of the intestinal mucosa. An early *in vitro* study with *C. difficile* 630 spores demonstrated that the exosporium layer was required for adherence to intestinal epithelial cells (IECs) and that this interaction was receptor(s) mediated, yet the identity of such receptors remains unclear (Paredes-Sabja and Sarker, 2012a). Recently, the collagen-like BclA1 spore glycoprotein of *C. difficile* 630 spores has been shown to affect the susceptibility of the host to colonization and infection (Phetcharaburanin et al., 2014). *In vivo* work, demonstrated that *C. difficile* forms biofilms and that vegetative cells bind to the intestinal mucus layer, however the presence of spores was not detected (Semenyuk et al., 2015). In addition, early studies demonstrated that the main cell surface layer protein (SLP) of the surface of *C. difficile* vegetative cells bounded to immobilized extracellular matrix molecules such as collagen I, thrombospondin, and vitronectin, but not to collagen IV, fibronectin or laminin (Calabi et al., 2002). *In vitro* experiments also demonstrated that the fibronectin binding protein, Fbp68, present in the cell surface mediated cell-adherence to Vero cells (Hennequin et al., 2003). However, whether *C. difficile* spores interact with these molecules remains unclear.

Two main strains have been used to study *C. difficile* spore-host interactions; strain 630 (PCR-ribotype 012) isolated from a patient with pseudomembranous colitis at a hospital in Zurich in 1982 (Sebaihia et al., 2006) and a representative strain from the Stoke Mandeville outbreak designated R20291 (McEllistrem et al., 2005). Most studies have used the strain 630 due to its easiness in genetic manipulation. However, the spores of 630 strain lack the hair-like projections of the outermost layer and have an exosporium-like layer that is ultrastructurally different than those observed in most epidemic *C. difficile* strains (Paredes-Sabja et al., 2014; Pizarro-Guajardo et al., 2016), suggesting that the observed phenotypes in 630 spores might not be reproducible in epidemic strain's spores possessing hair-like projections. A clear example of these phenotypic differences was elegantly demonstrated by Deakin et al. (2012) in a mouse model of infection recurrence, where the strain 630 was able to cause one episode of recurrence, contrasting with the multiple recurrent episodes caused by the epidemic strain R20291 (Deakin et al., 2012).

Despite the obvious importance of the outermost exosporium layer of *C. difficile* spores in host-pathogen interactions, there is a lack of experimental evidence on how relevant is this layer and its ultrastructural integrity in the interaction of *C. difficile* spores with components of the intestinal mucosa. In this context, we sought to characterize the adherence properties of spores from an epidemic *C. difficile* strain to components of the intestinal mucosa, such as intestinal epithelial cell lines, porcine stomach mucin, fibronectin and vitronectin. We observed that R20291 spores and vegetative cells exhibited a high degree of adherence to intestinal epithelial cells. Electron micrographs demonstrated a close proximity between the hair-like filamentous projections of the exosporium, the host's cell membrane and microvilli of IECs. Competitive-binding assay and trypsin digestion of Caco-2 cells, suggests that spore adherence might be receptor-mediated. Notably, spore affinity to bind to mucin-covered wells was lower than their vegetative morphotypes. Binding assays to fibronectin and vitronectin, indicate that these molecules bind in a concentration-dependent manner to the surface of *C. difficile* spores and that depending on the concentration they increase spore adherence to IECs. Notably, spores with an aberrantly assembled exosporium layer exhibited an increased adherence to IECs, mucin and fibronectin and vitronectin. It was also remarkable, that independent on the assembly state of the exosporium layer, only a fraction of the spores bind fibronectin and vitronectin to their surface. Collectively, these results provide insight into the affinity of *C. difficile* spores with components of the intestinal mucosa and the relevance of the exosporium layer in these interactions.

## Materials and methods

### Bacterial strains, cell lines, and culture conditions

*C. difficile* strain R20291 (RT027) is an epidemic strain that caused an outbreak and has been described elsewhere (McEllistrem et al., 2005; He et al., 2010). *C. difficile cdeC* mutant forms spores with an aberrantly assembled exosporium layer that lacks the hair-like projections and has been previously described (Barra-Carrasco et al., 2013). *C. difficile* strains were routinely grown under anaerobic conditions in a Bactron III-2 anaerobic chamber (Shellab, OR, U.S.A.) in 3.7% of Brain Heart Infusion supplemented with 0.5% yeast extract (BHIS) broth or on BHIS agar plates. In this study, intestinal epithelial Caco-2 and HT-29 cells, Vero cells which are derived from kidney epithelia of monkey and Hep2 cell-line which is a HeLa cell contaminant line were used to evaluate the adherence of *C. difficile*. Caco-2, HEp-2 and Vero cells were routinely grown in Dulbecco's modified Eagle's minimal essential medium (DMEM) (HyClone), while HT-29 cells were grown in RPMI medium (HyClone). All cell culture media were supplemented with 10% (vol/vol) fetal bovine serum (FBS) (HyClone), penicillin (100 U ml^−1^), and streptomycin (100 μ/ml). Caco-2 cells were kindly provided by Dr. Tulio Nuñez from the Laboratory of Cell Ageing at the Universidad de Chile; HEp-2, Vero and HT-29 cells were from an already existing stock in the Microbiota-Host Interaction and Clostridia Research Group at the Universidad Andrés Bello obtained from ATCC (U.S.A.).

### Spore preparations

Spore suspensions were prepared by plating a 1:500 dilution from an overnight culture onto 3% Trypticase Soy-0.5% yeast extract (TY) agar plates and incubated for 5 days at 37°C under anaerobic conditions. Spores were harvested with ice-cold sterile distilled water and purified with 50% Nicodenz as previously described (Sorg and Sonenshein, 2008). Spore suspensions were purified until they were >99% free of vegetative cells, sporulating cells and cell debris as determined by phase contrast microscopy and the number of spores/ml was quantified with a Neubauer Chamber (Sigma-Aldrich, U.S.A.) prior to use.

### Sonication of *C. difficile* spores

Spore suspensions of *C. difficile* strain *cdeC* (5 × 10^9^ spores/ml) were sonicated with ten pulses of 15 s separated by 3 min of cooling on ice-cold water as previously described (Barra-Carrasco et al., 2013; Escobar-Cortes et al., 2013).

### Spore adherence to epithelial cells

Spore adherence of R20291 spores epidemic strain to monolayers of cell-lines was measured as previously described (Paredes-Sabja and Sarker, 2012a). Briefly, Caco-2, HT-29, HEp-2 and Vero cells were seeded onto 96-well plates (5 × 10^5^ cells per well) and incubated until confluence. Caco-2 cells were further incubated for 2 and 8 days to obtain undifferentiated and differentiated monolayers, respectively, using previously described methods (Chantret et al., 1994). Differentiation of Caco-2 cells after 8 days was confirmed by immunofluorescence using Sucrose-Isomaltase as a marker for the appearance of the microvilli (Pinto, 1981) (data not shown) and transmission electron microscopy to visualize the presence of microvilli (Figure S1). Prior to infection, host cells were quantified for each experiment via trypan blue staining of trypsinized cells in a Neubauer chamber of a subset of wells. Monolayers were infected with *C. difficile* spores at a multiplicity of infection (MOI) of 10 in 40 μl of culture medium. In some experiments, undifferentiated and differentiated monolayers of Caco-2 cells were washed with Dulbecco's Phosphate-Buffered Saline (DPBS) to remove traces of FBS prior to pre-treatment with trypsin (0.05%) for 5 min before infection. In some experiments performed to evaluate the effect of FBS on spore adherence, R20291 spores (5 × 10^7^ spores sufficient for 10 wells) were pre-incubated for 1 h at 37°C in 40 μl of DMEM with 0, 5, or 10% (v/v) of FBS prior to infection. Spore-infected cells were incubated for 3 h at 37°C under aerobic conditions with 5% CO_2_, then unbound *C. difficile* spores were removed from spore-infected Caco-2 cells by washing three times with DPBS and lysed in 100 μl of 0.06% Triton X-100 in DPBS for 30 min at 37°C. Cell-spore lysates were serially diluted and aliquots plated onto BHI agar supplemented with 0.1% sodium taurocholate (BHIS-ST) (Himedia Laboratories Pvt. Ltd. Mumbai, India), and incubated under anaerobic conditions for 48 h at 37°C. For total *C. difficile* spore counts, spore-infected Caco-2 cells were directly lysed (no washing) with 100 μl of 0.06% Triton X-100 in DPBS for 30 min at 37°C and plated onto BHIS-ST agar plates, incubated anaerobically for 48 h at 37°C, and colony forming units (CFU) per ml counted. In some experiments, total cells per well were trypsinized and resuspended in DPBS and number of cells per well quantified with a Neubauer Chamber (Sigma-Aldrich, U.S.A.).

To measure adherence of *C. difficile* R20291 vegetative cells to undifferentiated and differentiated Caco-2 cells, monolayers were pre-reduced under anaerobic conditions for 2 h at 37°C and subsequently infected under anaerobic conditions for 3 h at 37°C at an MOI of 10. Non-adhered cells were rinsed off and total and adhered cells were quantified as described above. Viability of uninfected Caco-2 cells under anaerobic condition for 5 h was more than 95% as observed by trypan blue staining (data not shown).

The effect of fibronectin (Sigma-Aldrich, U.S.A) and vitronectin (Sigma-Aldrich, U.S.A) on the adherence of *C. difficile* spores to undifferentiated and differentiated monolayers of Caco-2 cells was evaluated by pre-incubating *C. difficile* spores (5 × 10^7^ spores) for 1 h at 37°C in 40 μl of serum free DMEM containing several concentrations of fibronectin or vitronectin (0, 0.5, 10, and 25 μg/ml). Monolayers were subsequently infected for 1 h at 37°C at an MOI of 10 with fibronectin- or vitronectin-treated *C. difficile* spores in the presence of 5% CO_2_, and the percentage of adherence was determined as described above.

### Mucin adherence assay

Ninety six-well plates were incubated overnight at 4°C with 0.04, 0.2, 0.5, and 1 mg/ml of mucin from pork stomach (Sigma-Aldrich, USA) as described elsewhere (Huang et al., 2011). Mucin-coated wells were then pre-reduced for 2 h under anaerobic conditions and infected with 40 μl of spore suspensions (5 × 10^6^ spores per well) or vegetative cells (5 × 10^6^ per well) for 3 h at 37°C under anaerobic condition, unbound spores were rinsed off with 3 washes with DPBS while bounded spores were subsequently removed with 100 μl of 0.06% Triton X-100 for 30 min at 37°C and plated onto BHIS-ST agar plates, incubated anaerobically for 48 h at 37°C, and CFU/ml counted. Total spore counts were determined from unwashed infected mucin-coated wells as described in the adherence assay.

### Alexa Fluor 488-labeling of *C. difficile* spores

Purified spores were labeled with Alexa Fluor 488 as previously described (Agerer et al., 2004; Paredes-Sabja and Sarker, 2012a).

### Competitive inhibition assay

Eight day-old monolayers of Caco-2 cells grown in 8-well culture slides (FD Falcon) were infected with 100 μl of DMEM containing unlabeled *C. difficile* R20291 spores at an MOI of 0, 10, 25, 50, 100 and 400 for 1 h at 37°C and washed three times with DPBS to remove unbound unlabeled *C. difficile* spores. Then Caco-2 monolayers were re-infected with Alexa Fluor 488 (green) labeled *C. difficile* spores at an MOI of 10 and incubated aerobically for 1 h at 37°C. Unbound fluorescently-labeled spores were washed three times with DPBS, fixed with paraformaldehyde and permeabilized with Triton X-100, stained for F-actin with phalloidin conjugated with Alexa 568 (red) and analyzed with a BX53 Olympus fluorescence microscope.

### Transmission electron microscopy

Six-well plates containing 2 and 8 day-old monolayers of Caco-2 cells were infected for 3 h at 37°C at an MOI of 10 with *C. difficile* R20291 spores and then unbound spores rinsed off as described above. Infected monolayers were fixed, embedded in spurs resin and prepared for transmission electron microscopy as previously described (Paredes-Sabja and Sarker, 2012b). Thin sections (90 nm) obtained with a microtome were placed on glow discharge carbon-coated grids and double lead stained with 2% uranyl acetate and lead citrate. Grids were analyzed with a Phillips Tecnai 12 Bio Twin electron microscope.

### Binding of fibronectin and vitronectin to *C. difficile* spores

To determine the binding dynamics and stoichiometry of fibronectin and vitronectin to *C. difficile* spores of epidemic R20291 strain we used modified protocols previously described (Xue et al., 2011). Briefly, spores (4 × 10^7^ spores) were incubated for 60 min at 37°C in 0.2% BSA containing various concentrations (1, 2.5, 5, 7.5, 10, 15, and 25 μg/ml) of purified human fibronectin and vitronectin (Sigma-Aldrich, USA). In some experiments spores of wild-type and *cdeC* mutant strains were used to evaluate the effect of the integrity of the exosporium layer on the relative binding of fibronectin and vitronectin. The spores were then washed three times with PBS, resuspended in 2X SDS-PAGE sample loading buffer, boiled twice for 5 min and electrophoresed on SDS-PAGE gels (12% acrylamide) using PageRuler Plus prestained Protein Ladder (Fermentas, U.S.A.). Proteins were transferred to a nitrocellulose membrane (Bio-Rad) and blocked for 1 h at room temperature with 2% bovine serum albumin (BSA)-Tris-buffered saline (TBS) (pH 7.4). Membranes were then probed with a 1:1000 dilution of rabbit anti-fibronectin and anti-vitronectin antibodies (Santa Cruz Biotechnologies, U.S.A.) for 1 h at room temperature, rinsed and then incubated at room temperature for 2 h with a 1:10,000 dilution of goat anti-rabbit IgG-horseradish peroxidase (HRP) conjugate (Invitrogen, Thermo Fisher Scientific, U.S.A.) in TBS with 1% BSA and 0.1% Tween 20. HRP activity was detected with a chemiluminescence detection system (Fotodyne Imaging system, U.S.A.) by using PicoMax sensitive chemiluminescence HRP substrate (RockLand Immunochemicals, U.S.A.).

Quantitative densitometry analysis of fibronectin and vitronectin binding to *C. difficile* spores was done using ImageJ software (http://imagej.nih.gov/ij/). The dissociation constant (*K*_D_) was calculated as follows: A = A_max_[protein]/ *K*_D_ + [protein], where A is the densitometric value of fibronectin or vitronectin at a given protein concentration, A_max_ is the maximum densitometric value for the Western blot data analysis (equilibrium), [protein] is the protein concentration in μg/ml and *K*_D_ is the dissociation equilibrium constant quantified as previously described (Xue et al., 2011).

### Solid-phase binding assay

Ninety six-well plate were coated overnight at 4°C with 100 μl/well containing 1.6 × 10^8^ spores or 1 μg of BSA in PBS (as a control). Then, the wells were washed five times with wash PBS containing 0.05% Tween 20 (PBST), blocked with 100 μl of PBS containing 2%-BSA and 0.05%-Tween 20 for 1 h at 37°C and subsequently incubated with 50 μl containing increased quantities of fibronectin or vitronectin (i.e., 0, 1, 10, 100, and 1000 nM) for 1 h at 37°C. Wells were then rinsed 5 times with 150 μl PBST prior to incubation with 50 μl of anti-fibronectin or anti-vitronectin (Santa Cruz Biotechnology, USA) at a 1:1000 dilution in PBST containing 1% BSA for 1 h at 37°C. Then, wells were rinsed 5 times with 150 μl of PBST and incubated for 1 h at 37°C with 50 μl of 1%-BSA in PBST containing a 1:5000 dilution of anti-rabbit (Rockland Inc. USA). After 5 washings with 150 μl of PBST, wells were rinsed once with 150 μl of 50 mM carbonate buffer (pH 9.6) prior to incubation with 50 μl of reaction buffer (17 mM citric acid, 65 mM potassium phosphate, 0.15%-hydrogen peroxide, 0.4%-θ-phenylenediamine for 20 min at room temperature. Reaction was stopped with 25 μl of 4.5N sulfuric acid. Binding was quantified by colorimetric detection at 450 nm in a 96-well plate reader (Infinite F50, Tecan, USA).

### Immunofluorescence of fibronectin and vitronectin bound to *C. difficile* spores

*C. difficile* wild-type and *cdeC* mutant spores (4 × 10^7^) were incubated for 60 min at 37°C in 0.2% BSA containing 2.5, 10, or 25 μg/ml of human fibronectin (Sigma-Aldrich, U.S.A.) and vitronectin (Sigma-Aldrich, USA). The spores were then washed three times with PBS and fixed in 3% paraformaldehyde (pH 7.4) for 20 min on poly-L-lysine-coated glass cover slides. Next, fixed spores were rinsed three times with PBS, blocked with 1% bovine serum albumin (BSA) for 30 min, and further incubated overnight at 4°C with 1:200 rabbit anti-fibronectin and anti-vitronectin antibodies (Santa Cruz Biotechnologies, USA). Then coverslips were incubated for 1 h at room temperature with 1:400 anti-rabbit IgG Alexa Fluor 568 conjugate (Invitrogen) in PBS with 1% BSA and washed three times with PBS and once with sterile distilled water. Samples were then dried at room temperature for 30 min, and then cover-slips were mounted using Dako Fluorescence Mounting medium (Dako North America) and sealed with nail polish. Samples were analyzed in an Olympus BX53 fluorescence microscope. A total of 300 spores were analyzed per experimental condition. Control experiments included spores with no fibronectin/vitronectin or with no primary antibodies, which did not yield fluorescence signal (data not shown). Using ImageJ (v1.48, NIH), an outline was drawn around 80 spores for each strain and area, the integrated density and the mean fluorescence measured, along with several adjacent background readings. Fluorescence intensity profiles were generated from the microscopy images using 3D Surface plotter function of ImageJ.

### Statistical analysis

Statistical analysis was performed using Statgraphics Centurion XVI (Statpoints Technologies, INC, USA). Student *t*-test was used for two-sample series comparison. One-way Analysis of Variance (ANOVA) multiple pairwise comparisons test with Bonferroni *post-hoc* tests were used to determine statistical significance. Mann–Whitney *U*-test was performed in Fluorescence intensity comparison between wild-type and *cdeC* spores.

## Results

### Adherence of spores of *C. difficile* epidemic strain R20291 to intestinal epithelial cells

**Figure 1:**
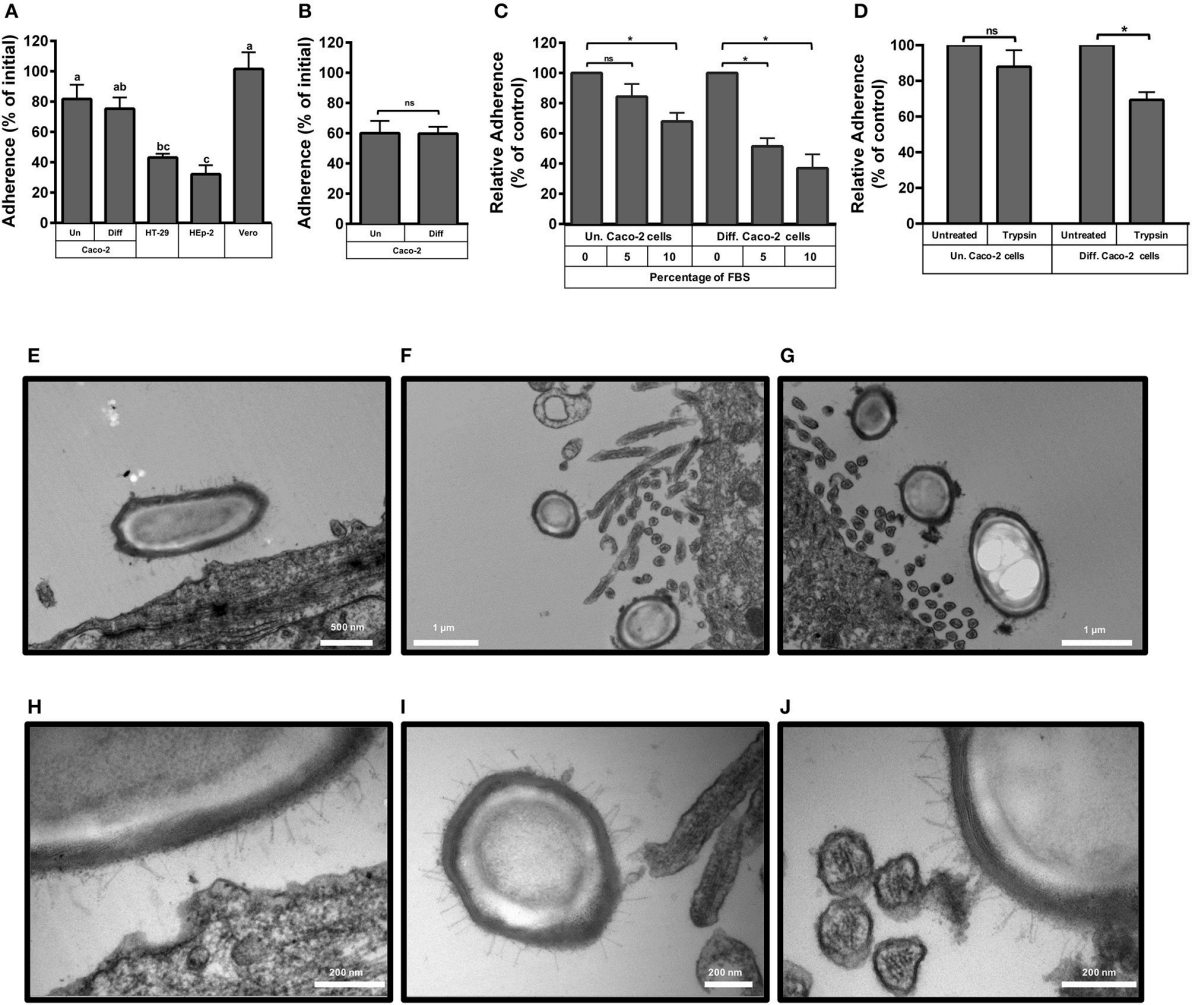
**Adherence of ***C. difficile*** epidemic R20291 spores to intestinal epithelial cell lines. (A)** Monolayers of undifferentiated (Un) and differentiated (Diff) Caco-2, HT-29, Hep-2 and Vero cells were infected at a MOI of 10 with *C. difficile* R20291 spores for 3 h at 37°C and adhered spores were determined as described in the Methods section. Pre-reduced monolayers of undifferentiated (Un) and differentiated (Diff) Caco-2 cells were infected for 3 h at 37°C under anaerobic conditions with an MOI of 10 of *C. difficile* R20291 vegetative cells and the percentage of adherence was determined as described in the Method section. **(B)** Pre-reduced monolayers of undifferentiated (Un) and differentiated (Diff) Caco-2 cells were infected at an MOI of 10 with vegetative cells of *C. difficile* R20291 strain for 3 h at 37°C under anaerobic conditions and the percentage of adherence was determined as described in the Method section. **(C)** The effect of fetal bovine serum (FBS) concentration on the adherence of *C. difficile* R20291 spores to monolayers of undifferentiated (Un) and differentiated (Diff) Caco-2 cells was determined by pre-incubating *C. difficile* spore suspensions with 0, 5, or 10% FBS prior to infection at an MOI of 10 for 3 h at 37°C as described in the Method section. **(D)** Effect of trypsin treatment of Caco-2 cells on spore adherence. The presence of putative cellular receptors was determined by trypsin-treating monolayers of undifferentiated (Un.) or differentiated (Diff.) Caco-2 cells for 5 min prior to infection with *C. difficile* spores at an MOI of 10 for 3 h at 37°C, as described in the Method section. Data represents the average of three independent experiments and error bars are standard error of the mean. In **(A)**, similar letters indicate no significant difference, while different letters indicate statistical difference (ANOVA; Bonferroni post-test; *P* < 0.05) between cell lines. Asterisks (^*^) in **(B–D)** denote statistical differences with a *P* < 0.01; ns, not significant. **(E–J)** Transmission electron micrographs of infected monolayers of Caco-2 cells. Monolayers of undifferentiated **(E,H)** and differentiated **(F,G,I,J)** Caco-2 cells were infected for 3 h at an MOI of 10 with epidemic spores of *C. difficile* strain R20291, unbound spores were rinsed off and infected monolayers were processed for transmission electron microscopy as described in the Method section. **(H–J)** Are magnified micrographs from those shown in **(E–G)**, respectively. White scale bars are shown in each micrograph.

Preliminary experiments indicate that the maximum adherence (i.e., nearly 100% of the spores) of *C. difficile* R20291 spores to cell-lines was reached after 5 h of infection, thus, experiments were performed at 3 h to detect differences. Adherence of R20291 spores reached 82 and 75% of the spores in undifferentiated and differentiated Caco-2 cells, respectively (Figure 1A). In contrast, spore adherence to the intestinal cell line HT-29 and to HEp-2 cells only reached 43 and 32% of total spores, respectively (Figure 1A). Conversely, spore adherence to Vero cells reached 100%. Adherence of R20291 vegetative cells revealed that 60 and 59% of vegetative cells adhered to undifferentiated and differentiated Caco-2 cells, respectively (Figure 1B). Although the adherence of *C. difficile* vegetative cells cannot be compared to that of *C. difficile* spores because adherence experiments were done anaerobic and aerobically, respectively, the levels of adherence were high in both morphotypes (Figures 1A,B). These results demonstrate the easiness with which *C. difficile* spores bind to host cells after 3 h of infection.

We also evaluated if pre-blocking of *C. difficile* spores with fetal bovine serum (FBS) would affect spore adherence to Caco-2 cells. We observed that incubation of R20291 spores with 10%, but not 5%, of FBS significantly (*p* < 0.01) reduced spore adherence to undifferentiated cells by 32% (Figure 1C). Reduction of spore adherence was more evident upon pre-blocking spores with 5 and 10% FBS prior to infection of differentiated Caco-2 cells, reducing spore adherence to levels of 49 and 37% of total spores, respectively (Figure 1C). These results suggest that serum molecules might bind to the spore and mask spore ligands involved in adherence to Caco-2 cells.

To assess whether a possible host-cell receptor might be implicated in adherence of epidemic R20291 spores to IECs, undifferentiated and differentiated Caco-2 cells were trypsin treated prior to spore-infection. No significant decrease in spore-adherence was observed in trypsin-treated undifferentiated Caco-2 cells, but a decrease in 31% in spore adherence was observed in trypsin-treated differentiated Caco-2 cells (Figure 1D), suggesting that host cell surface proteins present in differentiated Caco-2 cells might be involved in the adherence of R20291 spores.

### Hair-like extensions of the exosporium-like layer of R20291 spores are in close proximity with the host cell membrane and apical microvilli

To gain more insight into the interaction between *C. difficile* R20291 spores and IECs, transmission electron micrographs (TEM) of spore-infected monolayers of undifferentiated and differentiated Caco-2 were analyzed. TEM images of infected undifferentiated monolayers of Caco-2 cells demonstrate that R20291 spores are in close proximity with the cellular membrane (Figure 1E), and that this might be promoted by the hair-like extensions of the exosporium-like layer of *C. difficile* spores and an apparent foci formed in the cellular membrane (Figure 1H). TEM images of infected differentiated monolayers of Caco-2 cells suggest that the hair-like extensions from the exosporium-like layer of R20291 spores come in close proximity to the apical microvilli (Figures 1F,G). Magnified TEM images also provide evidence that these hair-like extensions are adjacent with either the tip (Figure 1I) or the lateral surface of the microvilli (Figure 1J). Collectively, these results suggest that *C. difficile* spores become in close proximity with IECs through the hair-like extensions of the exosporium-like layer of epidemic R20291 spores, presumably formed by the exosporium collagen like BclA glycoproteins, and the cellular membrane or the microvilli.

### Specificity of the adherence of epidemic *C. difficile* R20291 spores to intestinal epithelial cells

**Figure 2:**
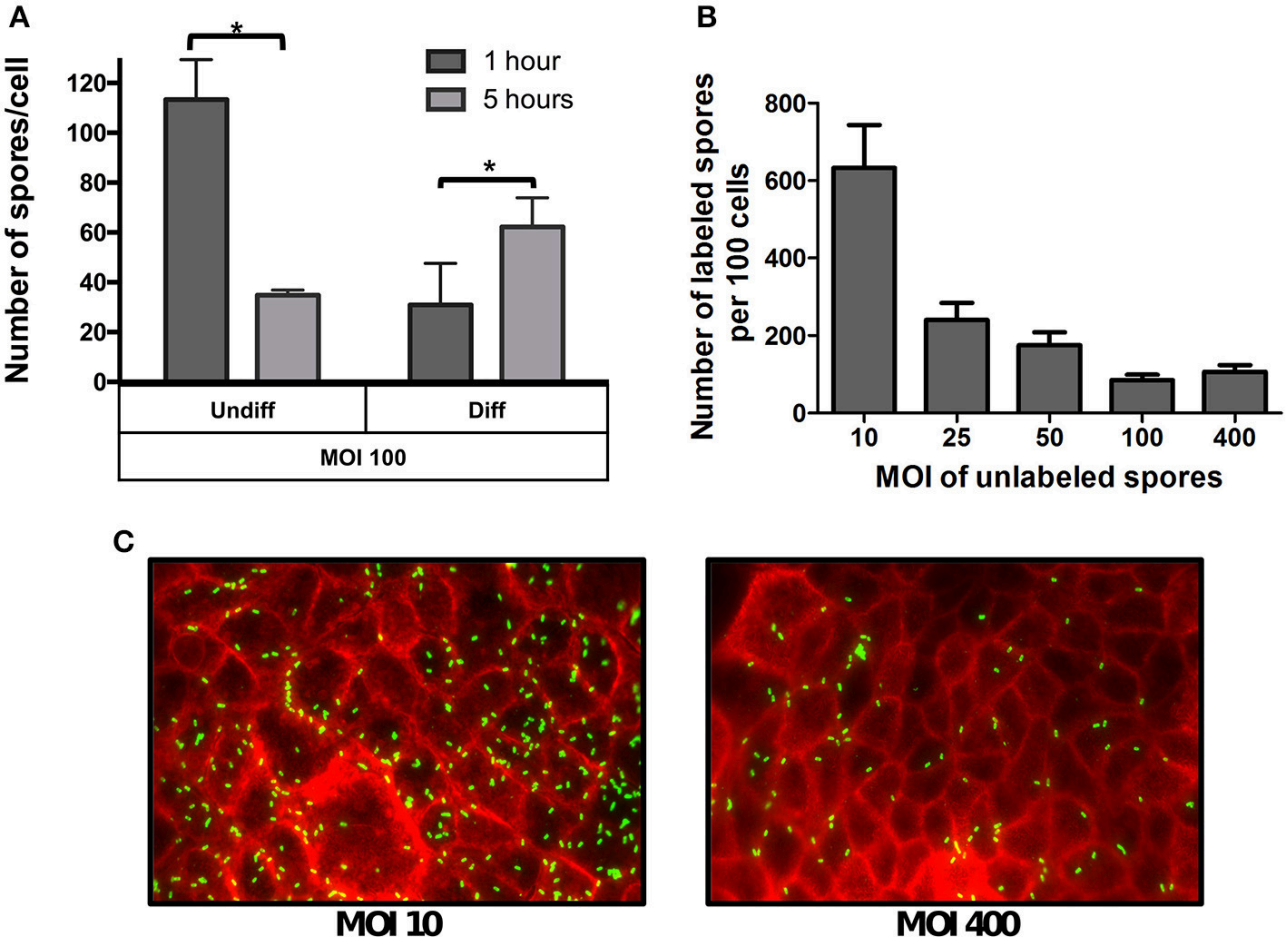
**Effect of the multiplicity of infection on spore adherence to intestinal epithelial cells. (A)** Monolayers of undifferentiated (Undiff) and differentiated (Diff) Caco-2 cells were infected at multiplicity of infection (MOI) of 100 for 1 (dark gray bars) or 5 h (light gray bars), respectively. Unbound spores were rinsed off and, total cells and adhered spores were determined as described in the Method section. **(B)** Competitive binding of unlabeled and fluorescently labeled *C. difficile* spores. Monolayers of differentiated Caco-2 cells were infected with different MOIs of unlabeled *C. difficile* 630 spores for 3 h. Then unbound spores were rinsed off, and subsequently infected for 3 h at an MOI of 10 with fluorescently labeled *C. difficile* spores. Unbound fluorescently labeled spores were rinsed off and subsequently processed and the number of fluorescently labeled analyzed by fluorescent microscopy. **(C)** Representative fluorescence micrographs of adhered fluorescently-labeled *C. difficile* spores (green) to monolayers pre-infected at an MOI of 10 and 400 with unlabeled spores. F-actin was stained with phalloidin conjugated with Alexa 568 (red). Data represents the mean of three biological experiments and error bars are standard error of the mean. Asterisks (^*^) denote statistical differences with a *P* < 0.01.

Given that the phenotypic differences in microvilli expression between undifferentiated and differentiated Caco-2 cells might affect the specificity of spore adherence, we quantitatively assess the specificity of spore-binding to undifferentiated and differentiated Caco-2 cells by infecting cells with a saturated level of spores (i.e., MOI of 100) for 1 and 5 h of infection. We reasoned that adherence at 1 h of infection would be reflective of non-specific binding, which would progress to specific binding after 5 h of infection due to the stabilization of putative spore-ligand(s) and host cellular receptor(s) interaction(s). Notably, infection of undifferentiated Caco-2 cells with MOI of 100, revealed high spore-adherence (110 ± 6 spores/cell) 1 h post infection, but a subsequent decrease to 35 ± 2 spores per cell after 5 h of infection (Figure 2A), suggesting that R20291 spores were initially adsorbed to IECs in a non-specific manner but only a fraction of the spores remained attached after 5 h of infection in a more specific-manner to Caco-2 cells. Interestingly, a different binding dynamic was observed when differentiated monolayers of Caco-2 cells were infected at the same MOI, since spore adherence increased from 31 to 62 spores per cell after 1 and 5 h of infection, respectively (Figure 2A).

The different spore binding dynamics between undifferentiated and differentiated Caco-2 cells when infected at a MOI of 100, suggests that spore adherence to differentiated Caco-2 cells might be mediated by host cell- receptor, and that these putative receptors might be located in the apical microvilli. To assess this, a competitive binding assay of *C. difficile* R20291 spores to Caco-2 cells was performed. Differentiated Caco-2 cells were first exposed to various MOIs (approximately 0, 10, 25, 50, 100, 400) of un-labeled R20291 spores for 3 h. Next, fluorescently labeled spores at a constant MOI of 10 were allowed to adhere for 3 h. Coverslips containing differentiated Caco-2 cells and spores were washed and analyzed by fluorescent microscopy. Notably, we observed an inverse correlation between the concentration of unlabeled spores and adherence of fluorescently labeled *C. difficile* R20291 spores (Figures 2B,C), suggesting that unlabeled spores might be competing for *C. difficile* spore-binding sites with fluorescently labeled spores.

### The integrity of the exosporium-like layer affects spore-adherence to IECs

**Figure 3:**
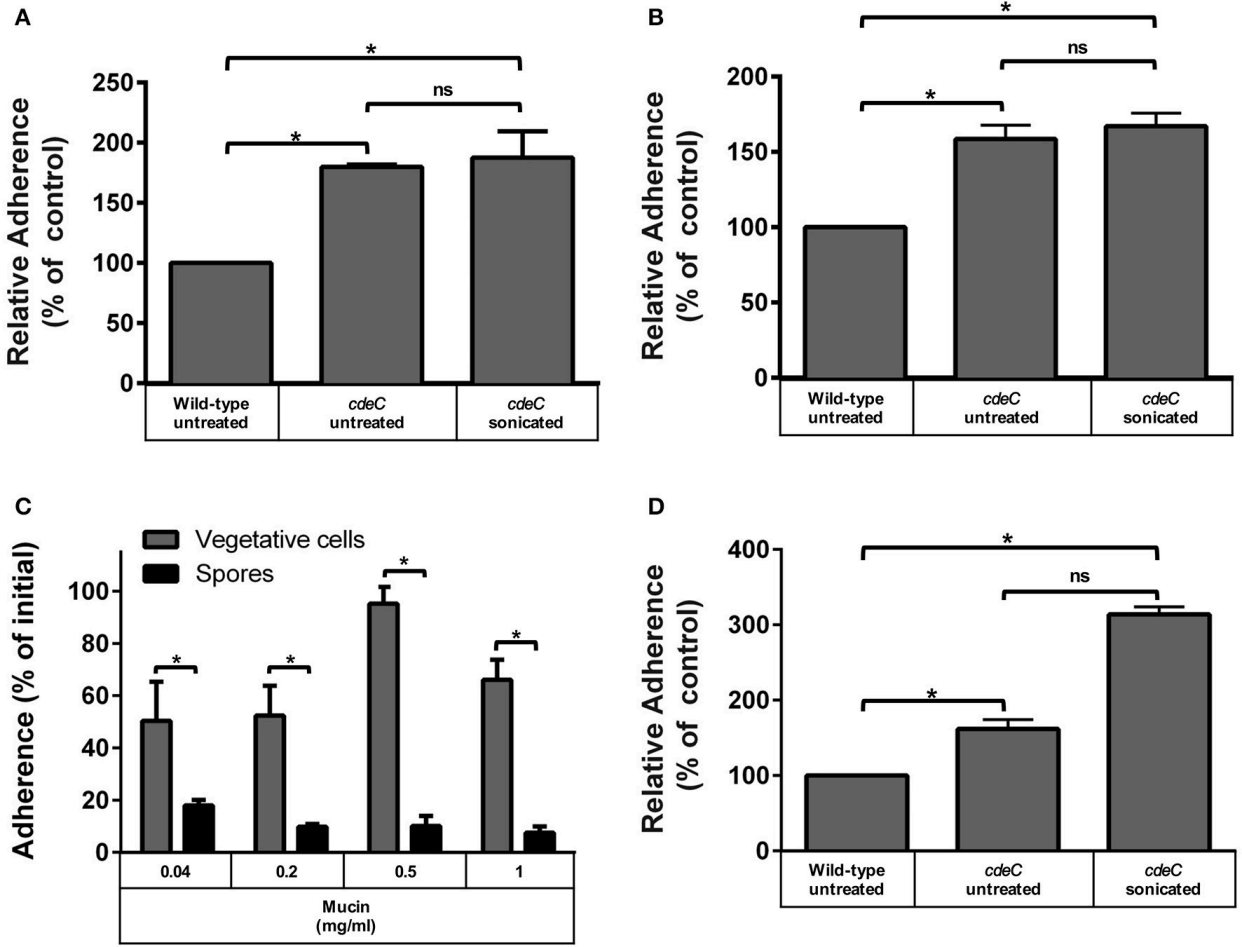
**Effect of the exosporium integrity on the tropism of ***C. difficile*** spores to intestinal epithelial cells and mucin**. Monolayers of undifferentiated **(A)** and differentiated **(B)** Caco-2 cells, were infected for 1 h at an MOI of 10 with *C. difficile* wild-type, untreated or sonicated *cdeC* mutant spores, unbound spores were rinsed off and adhered spores were quantified as described in the Method section. Data in **(A,B)** is expressed relative to the control which corresponds to the percentage of adherence of wild-type spores. Values are the mean of three independent experiments and error bars are standard error from the means. Asterisks (^*^) denote statistical differences with a *P* < 0.01; ns, not significant. **(C)** Adherence of *C. difficile* spores to mucin. The adherence of *C. difficile* vegetative cells (gray bars) and spores (black bars) to mucin was evaluated by incubating *C. difficile* vegetative cells or spores for 3 h at 37°C in wells containing different concentrations of mucin and adhered spores were determined as described in the Method section. Data corresponds to the average of three independent experiments and error bars are standard error of the mean. Asterisks (^*^) denote statistical differences with a *P* < 0.01 (Student's *t*-test). **(D)** *C. difficile* spores with a deficiently assembled exosporium layer exhibit increased adherence to mucin. Mucin-containing wells were infected with *C. difficile* wild-type spores, untreated or sonicated *cdeC* mutant spores, unbound spores were rinsed off and adhered spores were quantified as described in the Method section. Data is expressed relative to the control which is the percentage of adherence of wild-type spores. Data is the mean of three independent experiments and error bars are standard error from the means. Asterisks (^*^) denote statistical differences with a *P* < 0.01; ns, not significant (Student's *t*-test).

Since the aforementioned results suggest that spore-adherence to IECs is mediated by host cell-receptor, and might involve the filamentous extensions of the outermost layer of *C. difficile* spores, we hypothesized that the integrity of the exosporium-like layer is essential for adherence to IECs. To test this, we used spores of an otherwise isogenic *cdeC* mutant of *C. difficile* epidemic strain R20291, which albeit has a similar sporulation and germination phenotypes as wild-type spores, it has a deficiently assembled outermost layer that lacks the hair-like extensions (Barra-Carrasco et al., 2013). Strikingly, *cdeC* spores exhibited higher adherence to Caco-2 cells relative to wild-type spores when undifferentiated (79%; Figure 3A) and differentiated (58%; Figure 3B) monolayers of Caco-2 cells were infected. Therefore, to evaluate whether this increased adherence was attributed to remnants of the aberrantly assembled exosporium-like layer, *cdeC* spores were sonicated to ensure complete removal of this outermost layer, which was confirmed by a reduction in hydrophobicity to less than 3%, indicating complete exosporium removal (Escobar-Cortes et al., 2013). Notably, sonicated *cdeC* spores exhibited 87 and 67% more adherence than wild-type spores, but a similar extent of adherence as untreated *cdeC* spores to undifferentiated and differentiated Caco-2 cells, respectively (Figures 3A,B). Altogether, these results indicate that a correct assembly of the exosporium-like layer contributes to a selective adherence of *C. difficile* spores to IECs.

### Low mucin-adherence of *C. difficile* spores is attributed to a correctly assembled outermost layer

Despite recent evidence that *C. difficile* forms biofilm communities at the intestinal mucosa interface (Semenyuk et al., 2015), the affinity of its vegetative and spore morphotypes to mucin has not been evaluated. Therefore, wells covered with porcine stomach mucin were incubated with *C. difficile* R20291 vegetative cells or spores under anaerobic conditions to determine their binding affinity to mucin. Vegetative cells adhered to a high extent (i.e., ~50%) in presence of low levels of mucin (i.e., 0.04 and 0.2 mg/ml) (Figure 3C). A significant (*p* < 0.01) increase in adherence of vegetative cells to mucin was observed at 0.5 mg/ml, which decreased when wells were covered with 1 mg/ml (Figure 3C). Notably, R20291 spores had significantly lower adherence than vegetative cells to mucin, and the extent of adherence decreased in a concentration dependent manner (Figure 3C). These results suggest a differential tropism between vegetative cells and spores to the mucin layer. To determine if the integrity of the outermost layer was also required for a decreased adherence to mucin, experiments were repeated in mucin-covered wells with *cdeC* spores. Remarkably, *cdeC* spores had significantly higher adherence to mucin than wild-type spores (Figure 3D), which increased 3-fold upon complete removal of the remnants of the exosporium layer by sonication (Figure 3D). These results suggest that an aberrantly assembled exosporium layer will contribute to a higher adherence to the mucin.

### Binding of fibronectin and vitronectin to *C. difficile* spores increases adherence of spores to IECs

**Figure 4:**
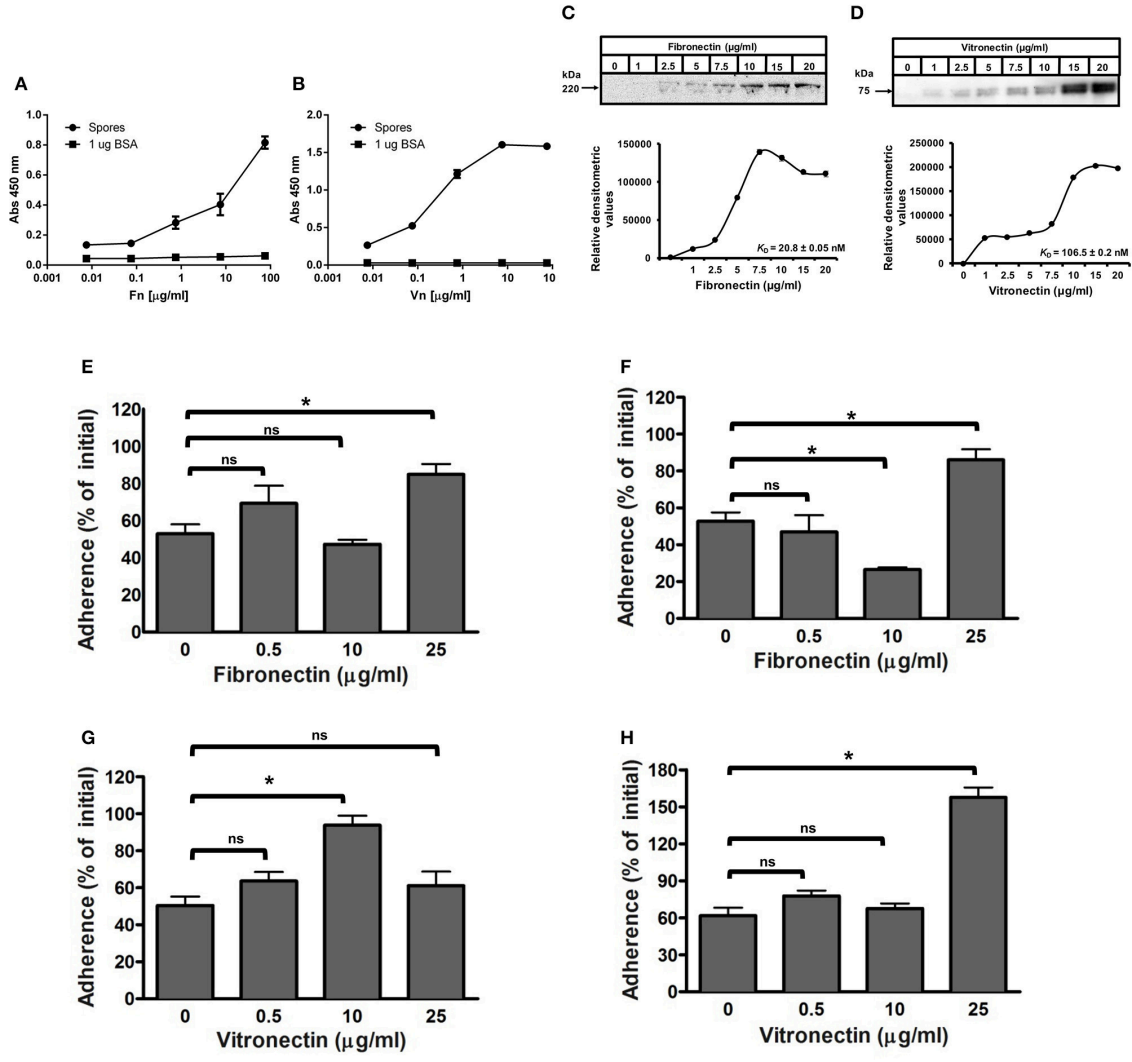
**Effect of binding of fibronectin and vitronectin to ***C. difficile*** R20291 spores in the adherence to intestinal epithelial cells. (A,B)** Solid phase binding affinity of fibronectin **(A)** and vitronectin **(B)** to *C. difficile* spores was assessed in spore- (black circles) or BSA-coated wells (black squares) as a control. Coated wells were then incubated with various concentrations of fibronectin (Fn) and vitronectin (Vn) for 1 h at 37°C and bound protein was detected as outlined in the Method section. The binding affinity of fibronectin **(C)** and vitronectin **(D)** to *C. difficile* wild-type R20291 spores was determined by incubating 4 × 10^7^ spores with various concentrations of fibronectin and vitronectin for 1 h at 37°C prior to resolving bounded molecules by SDS-PAGE gels, followed by Western blotting using fibronectin and vitronectin antibodies. Relative densitometric analysis of bounded fibronectin **(C)** and vitronectin **(D)** was performed to estimate the dissociation constant (*K*_*D*_) as described in the Method section using the background noise of 0 μg/ml of fibronectin and vitronectin as the reference values. Representative Western blots from three independent experiments are shown in **(A,B)**. Data represents the average of three independent experiments. **(E–H)** Effect of fibronectin and vitronectin on the adherence of *C. difficile* spores to intestinal epithelial cells. *C. difficile* spores were preincubated with various concentrations of fibronectin **(E,F)** and vitronectin **(G,H)** for 1 h at 37°C prior to infecting monolayers of undifferentiated **(E,F)** and differentiated **(G,H)** Caco-2 cells for 1 h at 37°C. Unbound spores were rinsed off and the percentage of adhered spores was calculated as described in the Method section. Data is the average of three independent experiments and error bars represents standard error of the mean. Student's *t*-test was used to compare the effect of fibronectin and vitronectin relative to the control. Asterisks (^*^) denote statistical difference with *P* < 0.01, respectively; ns, not significant (Student's *t*-test).

The extracellular matrix glycoproteins, fibronectin and vitronectin, have been used by numerous pathogenic bacteria as molecular bridges to bind to host surfaces and establish enteric infections (Singh et al., 2010; Henderson et al., 2011), yet their interaction with *C. difficile* spores remains unclear. To investigate the binding of fibronectin and vitronectin to R20291 spores, we used an adapted solid-phase ligand binding assay with R20291 spore-covered wells incubated with increasing concentrations of fibronectin and vitronectin; as a negative control, wells were coated with BSA. Fibronectin and vitronectin binding activity was evident in *C. difficile* spore-coated wells (Figures 4A,B). The specificity of these interactions was demonstrated in that the binding was dose dependent, suggesting that fibronectin and vitronectin have affinity to R20291 spores (Figures 4A,B). Additionally, we reasoned that to assert the concentrations of fibronectin and vitronectin that bind to *C. difficile* spores useful for spore-adherence assays, the binding of fibronectin and vitronectin was assessed by an alternative method; spores were first incubated with fibronectin and vitronectin in solution, harvested, washed and the exosporium extracts were electrophoresed and the amount of fibronectin and vitronectin detected by Western blot analysis (Xue et al., 2011). Then, the saturation of binding and the dissociation equilibrium constants (*K*_D_) of these molecules to the surface of R20291 spores was determined as described in the Methods section. A dose dependent and saturable binding was observed when increasing concentrations of fibronectin were allowed to bind to 4 × 10^7^ spores (Figure 4C). Saturation of fibronectin was observed at 10 μg/ml and based on the densitometry data the calculated *K*_D_ for fibronectin was of 20.8 nM (Figure 4C). Analysis of the binding of vitronectin to *C. difficile* spores gave a different binding dynamic, with two apparent binding sites; based on the densitometry data the maximum calculated *K*_D_ for vitronectin binding was estimated to be 106.5 nM (Figure 4D). Collectively, these results suggest that spore ligands might be implicated in binding of fibronectin and vitronectin.

Considering the results obtained from the binding assay, we assessed whether the binding of fibronectin and vitronectin to R20291 spores increase spore adherence to IECs, presumably by acting as a bridging molecule between spores and a specific αβ integrin receptor in IECs. Remarkably, a significant increase in spore adherence to Caco-2 cells was only observed when *C. difficile* spores were incubated with 25 μg/ml of fibronectin, increasing spore adherence from 53 to 85 and 53 to 86% to undifferentiated and differentiated monolayers, respectively (Figures 4E,F). In addition, pre-treatment of R20291 spores with 10 μg/ml of fibronectin decreased spore adherence to differentiated Caco-2 cells from 53% (control) to 27% (Figure 4F). Vitronectin also affected differently spore adherence to IECs since a significant increase in adherence of spores to undifferentiated Caco-2 cells from 50% (control) to 94% was detectable upon treatment of spores with 10 μg/ml of vitronectin (Figure 4G). However, spore adherence decreased to 61% upon treatment of spores with 25 μg/ml of vitronectin prior to infection (Figure 4G). Spore adherence to differentiated monolayers of Caco-2 cells was only increased when spores were treated with 25 μg/ml of vitronectin, increasing adherence from 61% (control) to 158% (Figure 4H). Taken together, these results indicate that, fibronectin and vitronectin might act as molecular bridging molecules by promoting adherence to IECs depending on their concentration.

### The integrity of the outermost layer affects binding of *C. difficile* spores to fibronectin and vitronectin

**Figure 5:**
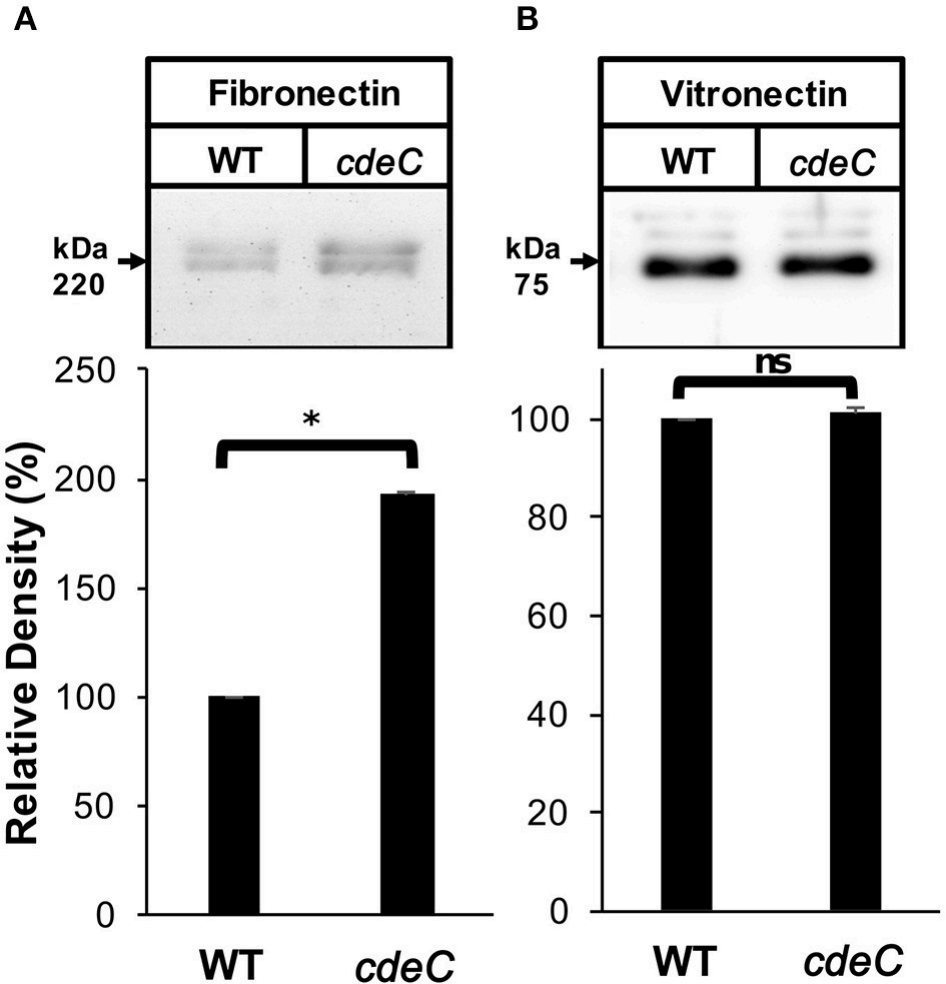
**Effect of the exosporium integrity on the binding of fibronectin and vitronectin to R20291 spores**. Wild-type (WT) and *cdeC* spores were pre-incubated with 10 μg/ml of fibronectin **(A)** or vitronectin **(B)** for 1 h at 37°C. Unbound fibronectin and vitronectin were rinsed off prior to resolving bounded molecules by SDS-PAGE gels, followed by Western blotting using fibronectin and vitronectin antibodies, as described in the Method section. Arrow indicates the molecular weight of fibronectin and vitronectin. Representative Western blots from three independent experiments are shown. Data is the average of three independent experiments and error bars represent standard error of the mean. Asterisks (^*^) denotes statistical difference at *P* < 0.01%; ns, not significant (Student's *t*-test).

We next evaluated whether the integrity of the exosporium layer is required for binding of fibronectin and vitronectin to *C. difficile* spores by analyzing the amount of fibronectin and vitronectin bound to the spore surface. Spores were incubated with 10 μg/ml of fibronectin and vitronectin for 1 h at 37°C and bound fibronectin and vitronectin were quantified by Western blot analysis of exosporium extracts. This concentration was selected to evaluate whether *cdeC* spores would bind more or less glycoproteins to their surface. We found that *C. difficile cdeC* spores bound 92% more fibronectin than wild-type spores (Figure 5A). By contrast, no significant increase in vitronectin-binding to *cdeC* spores, relative to wild-type spores, was detectable (Figure 5B). These results suggest that a correctly assembled exosporium layer decreases the amount of fibronectin, but not vitronectin, bound to the surface of *C. difficile* spores.

**Figure 6:**
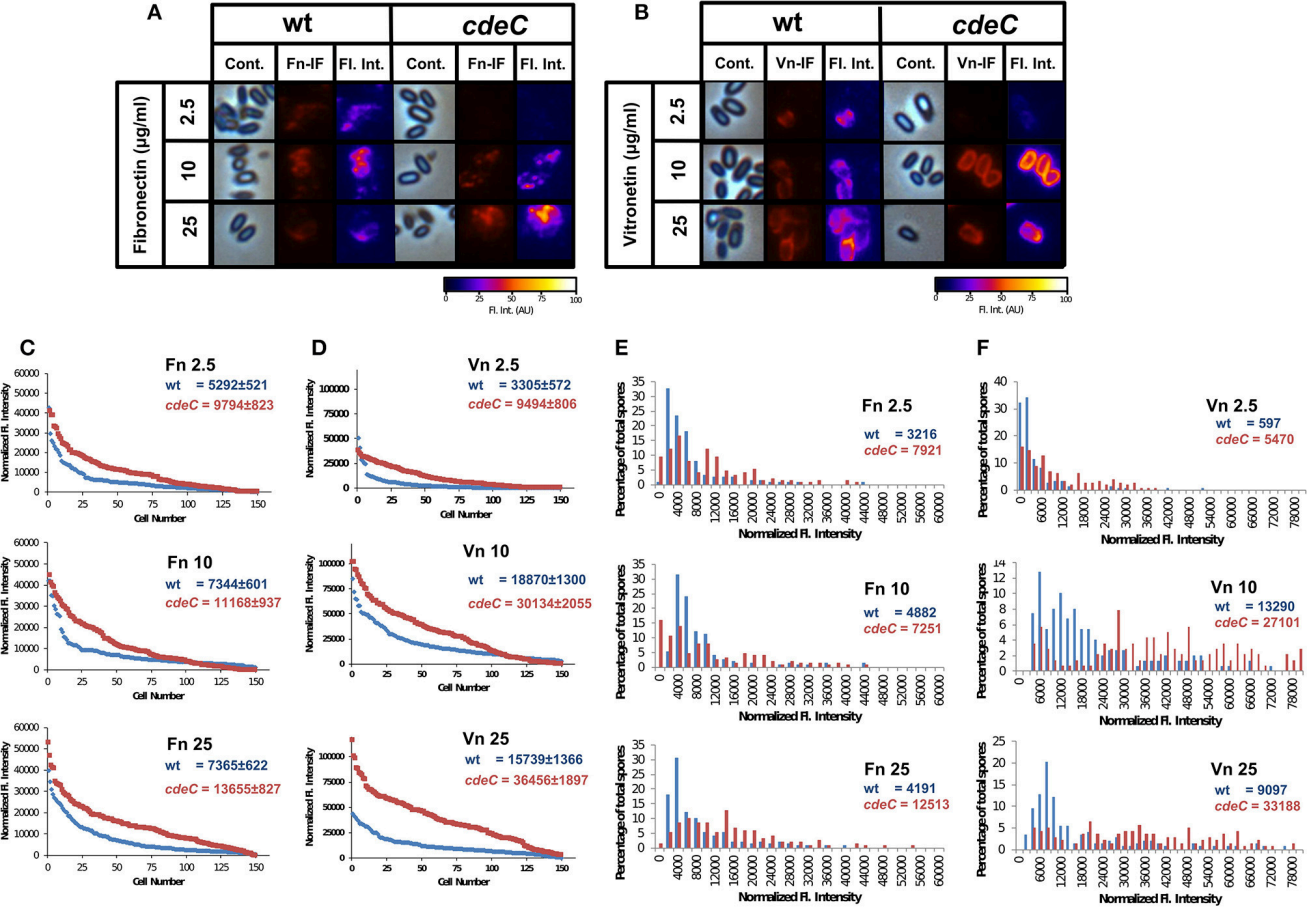
**Variability in the binding of fibronectin and vitronectin to ***C. difficile*** spores. (A,B)** *C. difficile* spores of wild-type (wt) and *cdeC* strains were incubated with various concentrations of fibronectin **(A)** and vitronectin **(B)** for 1 h at 37°C, centrifuge and stained by immunofluorescence for fibronectin and vitronectin as described in the Method section. Representative phase contrast (Cont.) and fibronectin- (Fn-IF) and vitronectin-specific (Vn-IF) immunofluorescence micrographs are shown for wild-type and *cdeC* spores. Fluorescence intensity (Fl. Int.) profiles of wild-type and *cdeC* mutant spores were provided by fluorescence microscopy images using the 3D Surface plotter function of ImageJ (http://imagej.nih.gov/ij/). **(C,D)** Quantitative analysis of the fluorescence (Fl.) intensity in spores of wild-type (blue line) and *cdeC* mutant (red line) incubated with different concentrations (i.e., 2.5, 10, and 25 μg/ml) of fibronectin (Fn) **(C)** or vitronectin (Vn) **(D)**. The values shown in the graphs are the average ± standard error of the fluorescence intensity from 150 spores from wild-type (wt) and *cdeC* strain. **(E,F)** Distribution of the fluorescence intensity of wild-type (wt, blue bars) and *cdeC* spores (*cdeC*, red bars) incubated with various concentrations (i.e., 2.5, 10, and 25 μg/ml) of fibronectin (Fn) **(E)** or vitronectin (Vn) **(F)**. The median of the fluorescence intensity of wild-type and *cdeC* spores is shown in each panel. The data shown in **(C–F)** are from one experiment that is representative of three independent experiments.

Recently, Pizarro-Guajardo et al. (2016) demonstrated by transmission electron microscopy that two distinctive exosporium morphotypes (i.e., a thin- and a thick-exosporium layer) from a same batch of spores were detectable in various epidemic ribotypes, including R20291 (Pizarro-Guajardo et al., 2016). Therefore, we sought to evaluate whether fibronectin- and vitronectin-binding was homogenous in a spore population by indirect immunofluorescence. The effect on how an aberrantly-assembled exosporium layer affected the pattern of fibronectin- and vitronectin-binding to the spore surface was also evaluated. Analysis of the fluorescent micrographs of wild-type spores incubated with various concentrations of fibronectin revealed that only a fraction of the spores yielded fibronectin-specific fluorescence signal independently on the concentration of fibronectin used (Figures S2, S3A). The pattern of fibronectin-specific immunofluorescence in fibronectin-positive spores was not homogeneously distributed around the spores, with spots exhibiting higher fluorescence intensity at defined spots of the spore (Figure 6A). Upon analysis of fluorescent micrographs of *cdeC* spores, we observed a similar pattern as described for wild-type, but apparently a wider ring of fibronectin binding, presumably due to the aberrantly assembled exosporium layer (Figure 6A). Analysis of fluorescent micrographs revealed that the percentage of wild-type spores with visually positive fibronectin-specific immunofluorescent signal was 73, 80, and 86% upon incubation with 2.5, 10, and 25 μg/ml, respectively (analysis of 450 individual spores per treatment) (Figure S3A). Similar values of fibronectin-positive *cdeC* spores were observed (Figure S3A).

Upon analysis of the binding of vitronectin to wild-type spores by immunofluorescence (Figure S2), we observed that some wild-type and *cdeC* spores yielded vitronectin-specific fluorescence signal (Figure 6B). Independently of the concentration of vitronectin, those spores that were positive for anti-vitronectin antibodies, exhibited an immunofluorescence signal that homogeneously surrounded the entire spore (Figure 6B). A similar binding pattern of vitronectin was observed in spores with an aberrantly assembled exosporium layer (Figure 6B). Analysis of fluorescent micrographs revealed that the percentage of wild-type spores positive for vitronectin-specific immunofluorescent signal was 69, 83, and 90% of total spores upon incubation with 2.5, 10, and 25 μg/ml, respectively (analysis of 450 individual spores per treatment) (Figure S3B). Similar values of vitronectin-positive *cdeC* spores were observed (Figure S3B). Taken together, these results suggest that independent to the integrity of the exosporium layer, a fraction of the spores are capable of binding fibronectin and vitronectin to their surface.

To quantitatively analyze the effect of exosporium integrity in the binding of fibronectin and vitronectin to *C. difficile* spores, fibronectin- and vitronectin-specific fluorescence signal was quantified in wild-type and *cdeC* spores. Spores with an aberrantly assembled exosporium layer had a higher average fibronectin-specific fluorescence intensity than wild-type spores, which was 1.8-, 1.5-, and 1.9-fold higher than wild-type spores when incubated with 2.5, 10, and 25 μg/ml of fibronectin, respectively (Figure 6C). It is noteworthy that the fibronectin fluorescence intensity reached its maximum in wild-type and *cdeC* spores upon incubation with 10 and 25 μg/ml of fibronectin, respectively (Figure 6C). Analysis of the vitronectin-specific fluorescence intensity of wild-type and *cdeC* spores revealed, that independent to the concentration of vitronectin, spores with an aberrantly-assembled exosporium layer (*cdeC* spores) reached an average vitronectin-specific fluorescence intensity 1.7-, 1.6-, and 2.3-fold higher than that of wild-type spores. Similarly, as with fibronectin, the vitronectin-specific fluorescence intensity of wild-type and *cdeC* spores reached a maximum upon incubation of spores with 10 and 25 μg/ml of vitronectin (Figure 6D). These results suggest that, although fibronectin and vitronectin may bind to the spore surface, most of their ligands are hidden in a correctly assembled exosporium layer.

To gain more insight into the relevance of a correctly-assembled exosporium layer on the binding of fibronectin and vitronectin to *C. difficile* spores, histograms of the fluorescence intensity were analyzed. The distribution of the fibronectin-specific fluorescence intensity in wild-type spores remained skewed, independent of the concentration of fibronectin used (Figure 6E), with medians for fluorescence intensity of 3216, 4882, and 4191 upon incubation with 2.5, 10, and 25 μg/ml of fibronectin, respectively (Figure 6E). We found that, the distribution of the fibronectin-specific fluorescence intensity of *cdeC* spores was notoriously less skewed than that of wild-type spores, and the median fluorescence intensity increased 2.5-, 1.5-, and 3.0-fold relative to wild-type upon incubation with fibronectin (Figure 6E). The distribution of vitronectin-specific fluorescence intensity also exhibited a skewed distribution in wild-type spores, with medians of 597, 1390, and 9097 upon incubation with 2.5, 10, and 25 μg/ml, respectively (Figure 6F). The distribution of the vitronectin-specific fluorescence intensity in *cdeC* spores was only skewed upon incubation with 2.5 μg/ml of vitronectin, and exhibited a broad range binomial distribution upon incubation with 10 and 25 μg/ml of vitronectin (Figure 6F). Remarkably, the median of the fluorescence intensity was 9-, 2-, and 3.7-fold higher than in wild-type spores upon incubation with 2.5, 10, and 25 μg of vitronectin (Figure 6F). Collectively, these results suggest that: (i) the assembly of the exosporium hides binding sites for fibronectin and vitronectin, reducing the affinity of the spore to these glycoproteins; and (ii) in spores with an aberrantly assembled exosporium layer, these binding sites become available to exhibit an increased binding of these glycoproteins.

## Discussions

To the best of our knowledge, there are only a few studies that address how *C. difficile* spores interact with host tissue (Paredes-Sabja and Sarker, 2012a; Phetcharaburanin et al., 2014). Recent work on *C. difficile* spores and their interactions with host's molecules and/or the host have used strain 630 (Paredes-Sabja and Sarker, 2012a; Phetcharaburanin et al., 2014), which has been demonstrated to form spores with an outer layer that lacks hair-like projections and the typical bumps formed by unidentified electron dense material that underlines the hair-like projections, which are common features of several epidemic *C. difficile* ribotypes (Paredes-Sabja et al., 2014; Pizarro-Guajardo et al., 2016). This major ultrastructural observation supports the need to explore how spores of a representative epidemic strain, with outer layer with hair-like projections, interact with host components of the intestinal mucosa. In this context, the results in this work characterize the interactions of epidemic spores with host-components and how the exosporium affects these interactions. Although a drawback of this work is that most results are descriptive, they do highlight the impact of the exosporium layer on the tropism of *C. difficile* spores toward host components of the intestinal mucosa, providing a starting point for a wide range of studies to dissect the mechanism of *C. difficile* spore-host interactions.

Previous work with *C. difficile* 630 spores divulgated that *C. difficile* spores could adhere to IECs at relatively high levels in a receptor mediated manner (Paredes-Sabja and Sarker, 2012a), yet the identity of such a receptor has not been identified. In this work we observed that independent of the cell line, epidemic R20291 spores highly adhere to IECs. Interestingly, the nearly 100% of adherence observed in Vero cells suggests high expression of a putative host cell receptor(s) in comparison to Caco-2, HT-29 or HEp-2 cells. In addition, transmission electron micrographs showed that *C. difficile* R20291 spores and IECs come into close proximity, presumably by the interaction of the hair-like extensions and some unidentified receptor in the host-cell membrane of undifferentiated cells or in the apical microvilli of differentiated cells. We attempted to address this specificity with an adherence assay at saturating MOI (i.e., 100), and evidenced: (i) in undifferentiated cells, a rapid adsorption of the spores occurred within the first h of infection, leading to high levels of adherence, which decreased after 5 h of infection, presumably due to initial unspecific interaction attributed to Brownian motion and electrostatic interactions, which are rapidly lost by the lack of spore-specific receptors in undifferentiated IECs; (ii) in differentiated cells, a slow but steady increase in the adherence of *C. difficile* spores after 1 and 5 h, suggesting differences in the binding kinetics that might be receptor-mediated. This was supported by the competitive binding assay on differentiated Caco-2 cells and the trypsin-digestion of differentiated Caco-2 cells, suggesting that cellular receptor(s) are mediating adherence of epidemic R20291 spores to IECs. It is noteworthy that the source of Caco-2 cells used in this study differed from our previous work (Barra-Carrasco et al., 2013) and might account for the differences in the levels of spore adherence observed in this study vs. our previous work.

Adherence of vegetative cells of *C. difficile* has been previously analyzed by quantitative real-time PCR yielding low adherence levels (i.e., 0.6% of total cells) upon infection of monolayers of Caco-2 cells with a MOI of 1 (Dingle et al., 2010). Here we demonstrate that *C. difficile* vegetative cells also have high adherence to IECs, though using an MOI of 10. It was most remarkable to observe that *C. difficile* vegetative cells had a higher affinity to mucin than *C. difficile* spores. Although these experiments were performed with porcine stomach mucin, and not with Muc-2 which is mostly prominent in colonic mucosa (Johansson et al., 2011), the results suggest that the colonic mucosal-layers will be readily colonized by *C. difficile* vegetative cells rather than by spores, and that both morphotypes will have high affinity toward IECs. Further work on the dynamics of *C. difficile* spores and vegetative cells in the colonic mucosa will help address these questions.

Extracellular matrix molecules (i.e., fibronectin and vitronectin) are broadly used by bacterial pathogens to gain entry into the host since they regulate several processes and enzymes involved in extracellular matrix remodeling (Singh et al., 2010; Henderson et al., 2011). Fibronectin and vitronectin are multifunctional glycoproteins of the extracellular matrix and serve as a potential docking target for pathogenic bacteria to cellular tissues (Singh et al., 2010; Henderson et al., 2011). To the best of our knowledge, while binding of vitronectin to *C. difficile* has not been addressed, early studies with fibronectin provided evidence that *C. difficile* vegetative cells had affinity toward this molecule via a fibronectin binding protein, Fbp68 (Hennequin et al., 2003). Later work with a *fbp68*^−^ mutant background suggested that Fbp68 did not display a role in the pathogenesis of *C. difficile* in a mouse model of infection (Barketi-Klai et al., 2011). Our results demonstrate that *C. difficile* spores bind fibronectin and vitronectin to their surface and that depending on the concentration of these molecules, adherence to IECs may be favored. It is noteworthy that our results demonstrate that *C. difficile* spores bind to soluble fibronectin and vitronectin at levels found under physiological conditions (Tas et al., 2014). The binding dynamics of these molecules generated intriguing conclusions that suggest that while there might be one fibronectin binding sites, the spore surface might have two alternative binding sites for vitronectin. Moreover, our results also demonstrate that while there was a trend to increased spore adherence of *C. difficile* spores to undifferentiated and differentiated Caco-2 cells, pre-incubation of *C. difficile* spores with 10 μg/ml of fibronectin decreased the adherence levels. This became particularly significant in differentiated Caco-2 cells, suggesting that fibronectin might mask spore-ligand(s) that directly interact with cellular receptor(s) of differentiated Caco-2 cells. Clearly further work uniquely focused to understand the interaction of *C. difficile* spores to these molecules will uncover novel spore ligand(s) to host molecules and their implication *in vivo*.

The general concept is that vitronectin-integrin receptors are mainly present in the basolateral layer of epithelial cells, yet in undifferentiated epithelial cells apical expression of α_5_β_3_ has been reported (Bergmann et al., 2009). This might also be the case with undifferentiated Caco-2 cells used in this study. By contrast, Caco-2 cells have been shown to express the fibronectin-specific α_5_β_1_ integrin receptor at the apical membrane (Kolachala et al., 2007), yet whether the fibronectin- and vitronectin-enhanced adherence of *C. difficile* spores to Caco-2 cells observed in our work is due to these molecules acting as a molecular bridge between the spores and their specific αβ integrins receptors, is matter of further study. Another notable observation of this work was that only a fraction of the spores bound fibronectin and vitronectin to their surface, suggesting that fibronectin and vitronectin-ligand(s) might be present only in a fraction of the spores. This might correlate with the ability of *C. difficile* to produce two distinctive populations of spores, (i.e., those with a thick exosporium layer and those with a thin exosporium layer) (Pizarro-Guajardo et al., 2016). Further ongoing studies are expected to address the implications of these two different morphotypes in the pathogenesis of the infection.

The aforementioned electronic micrographs suggest that the hair-like filaments of the outermost exosporium layer might be involved in the interaction of *C. difficile* spores with host cells. To address the importance of the exosporium layer in the interaction of *C. difficile* spores and components of the intestinal mucosa, we used spores from a *cdeC* mutant background which have an aberrantly-assembled exosporium layer (Barra-Carrasco et al., 2013) and therefore differences in the behavior of these spores relative to wild-type would provide insight into the relevance of the exosporium integrity. In this context, the higher adherence of *cdeC* spores compared to wild-type spores to undifferentiated and differentiated Caco-2 cells and mucin suggests a controversial role of the exosporium layer in the adherence to host surfaces. It seems that this layer might contribute with specific-binding to unidentified cellular receptor(s), and upon removal of the outermost layer, spore adherence becomes non-specific as observed with *cdeC* spores. Unfortunately, although our experiments do not allow the asseveration of this hypothesis, as no competitive binding experiments were performed with *cdeC* spores, these observations raise the notion that the exosporium might contribute with selective binding to components of the intestinal mucosa, and with tropism of *C. difficile* spores toward epithelial cells rather than mucin. Removal of remnants of the exosporium layer by sonication did not improve the adherence of *cdeC* spores to IECs, but increased their adherence to mucin, suggesting that the spore coat might hold higher affinity to mucin than the exosporium layer, thus spores with a defective exosporium layer are likely to remain in the host while those that have an intact structure might be prone to be shed from the host. Further *in vivo* studies to evaluate whether the exosporium favors the release of spores to the environment in a healthy intestinal mucosa, vs. the persistence of *C. difficile* spores in a damaged mucosa would provide evidence to this notion. A possible weakness in this work is the lack of information of the proteomic composition of the aberrantly assembled exosporium layer of *cdeC* spores. However, the use of these spores highlights the relevance of the integrity of the exosporium layer, and we expect that further proteomic work will address many of the questions that arise from this work. It was also notable to observe that only a small fraction (i.e., 5–20%) of wild-type spores remained attached to the mucin-covered plates. It might be conceivable that those spores that remain attach to mucin-covered plates might have unique exosporium properties that correlate with one of the two exosporium morphotypes (i.e., thick or thin) recently identified (Pizarro-Guajardo et al., 2016). The notion that intact exosporium layer regulates the tropism of *C. difficile* spores with the intestinal mucosa is supported by the binding assays of *cdeC* spores to fibronectin and vitronectin; where immunofluorescence micrographs provided evidence that higher levels of fibronectin and vitronectin bounded to the surface of *cdeC* spores compared to wild-type spores, as well as by the lack of a skewed distribution as that observed in wild-type spores. These observations also conceive that ligand(s) specific for both molecules are hidden in a correctly assembled exosporium layer, and might become exposed during disintegration of the exosporium layer, facilitating the interaction of the spore with fibronectin and vitronectin, and therefore adherence to IECs. It was also remarkable that independent of the integrity of the exosporium, ~ one-third of the spores were negative for fibronectin- and vitronectin-specific immunofluorescence, observations that might correlate with the fact that ~ one-third of the R20291 spores have a thick exosporium layer (Pizarro-Guajardo et al., 2016). This supports the notion that some spores have fibronectin and vitronectin ligand(s), raising further questions on the implications of exosporium variability on the pathogenesis of *C. difficile*.

In summary, the integrity of the exosporium layer of *C. difficile* spores seems to regulate the tropism of the spores in the host, where an intact exosporium will favor spore shedding while those spores with a defectively assembled or partially degraded exosporium layer, will exhibit higher affinity to components of the intestinal mucosa. The exosporium layer of *C. difficile* spores, although considered a robust structure, can be removed by protease digestion (Escobar-Cortes et al., 2013) and lost during long term storage at ambient conditions (Pizarro-Guajardo et al., 2016). These observations conceive the hypothesis that ingested spores from the environment (i.e., that might have a damaged exosporium layer), would bind to mucin, presumably germinate and colonize in a susceptible host. During the course of the infection, the newly formed spores (i.e., with a correctly assembled exosporium layer) are likely to bind preferentially to toxin-mediated accessible IECs and/or be released to the environment, rather than remaining attached to the mucin layer. However, it is compelling to speculate that germinating spores, in which the exosporium is being degraded, are more adherent and might contribute in the maintenance of the infection. In light of the recent advances in the mouse model of CDI (Koenigsknecht et al., 2015), the *in vivo* implication of the integrity of the exosporium layer in persistent infection remains to be determined.

## Author contributions

Conceived and designed experiments: PM, CM, PC, DP. Performed experiments: PM, CM, PC. Analyzed data: PM, CM, PC, DP. Contributed with reagents/materials/analysis tools: MS, DP. Contributed writing the manuscript: FG, IC, JF, PR, SB, MS, DP.

### Conflict of interest statement

The authors declare that the research was conducted in the absence of any commercial or financial relationships that could be construed as a potential conflict of interest.

## Supplementary material

Figure S1 **Transmission electron micrographs of differentiated monolayers of Caco-2 cells**. Monolayers were processed for transmission electron microscopy as described in the Method section to observe the appearance of apical microvilli. Black scale bar is shown (5 μm).

Figure S2 **Fibronectin and vitronectin-specific immunofluorescence of ***C. difficile*** spores. (A,B)** *C. difficile* spores of wild-type (wt) and strain *cdeC* were incubated with various concentrations of fibronectin **(A)** and vitronectin **(B)** for 1 h at 37°C, centrifuge and stained by immunofluorescence for fibronectin and vitronectin as described in the Method section.

Figure S3 **Percentage of fibronectin- and vitronectin-specific immunofluorescent positive spores**. *C. difficile* wild-type (black bars) and *cdeC* spores were incubated with different concentrations of fibronectin **(A)** and vitronectin **(B)** and processed for indirect immunofluorescence as described in the Method section. Immunofluorescence micrographs were analyzed for the presence or absence of visual immunofluorescence signal. A total of 450 spores were counted. Bars represent the mean of three independent experiments and error bars represent standard error from the mean.
