# Supplementary figures and images for: Characterization of the Adherence of Clostridium difficile Spores: The Integrity of the Outermost Layer Affects Adherence Properties of Spores of the Epidemic Strain R20291 to Components of the Intestinal Mucosa
Source: Front Cell Infect Microbiol. 2016 Sep 22;6:99. doi: 10.3389/fcimb.2016.00099 (PMC5031699; doi:10.3389/fcimb.2016.00099)

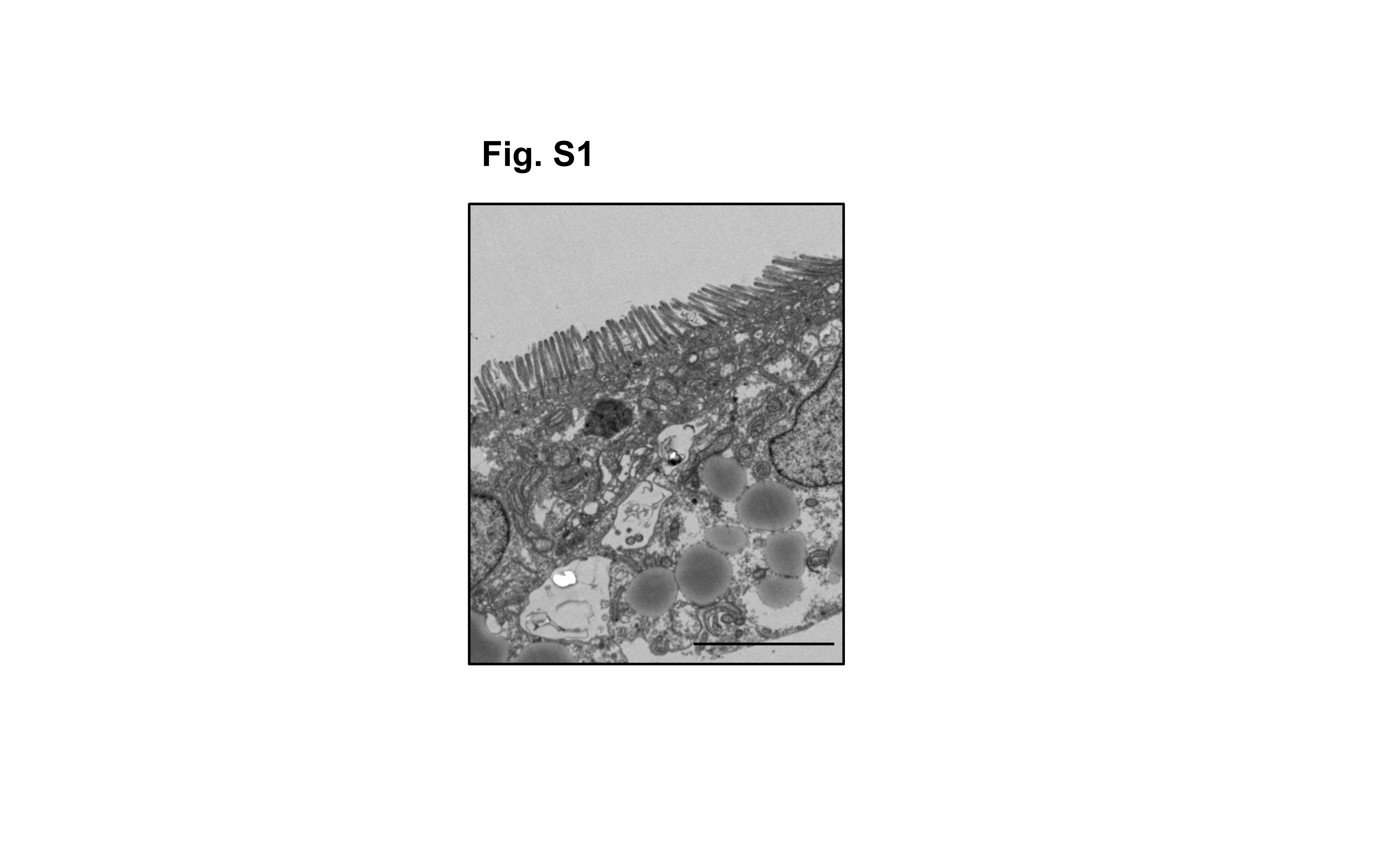

Supplement: Figure S1 — Transmission electron micrographs of differentiated monolayers of Caco-2 cells. Monolayers were processed for transmission electron microscopy as described in the Method section to observe the appearance of apical microvilli. Black scale bar is shown (5 μm). [file Image1.TIFF]

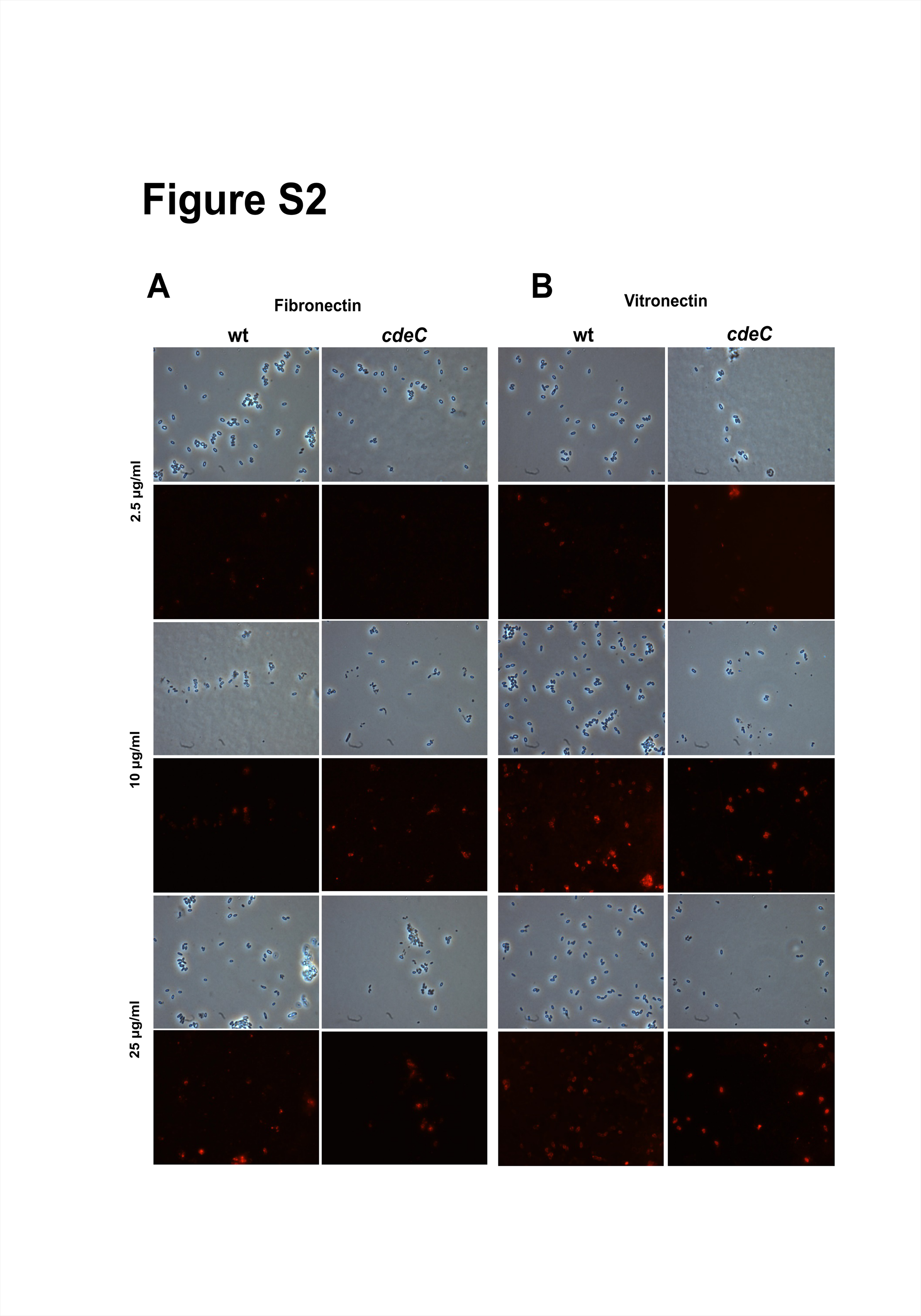

Supplement: Figure S2 — Fibronectin and vitronectin-specific immunofluorescence of C. difficile spores. (A,B) C. difficile spores of wild-type (wt) and strain cdeC were incubated with various concentrations of fibronectin (A) and vitronectin (B) for 1 h at 37°C, centrifuge and stained by immunofluorescence for fibronectin and vitronectin as described in the Method section. [file Image2.TIFF]

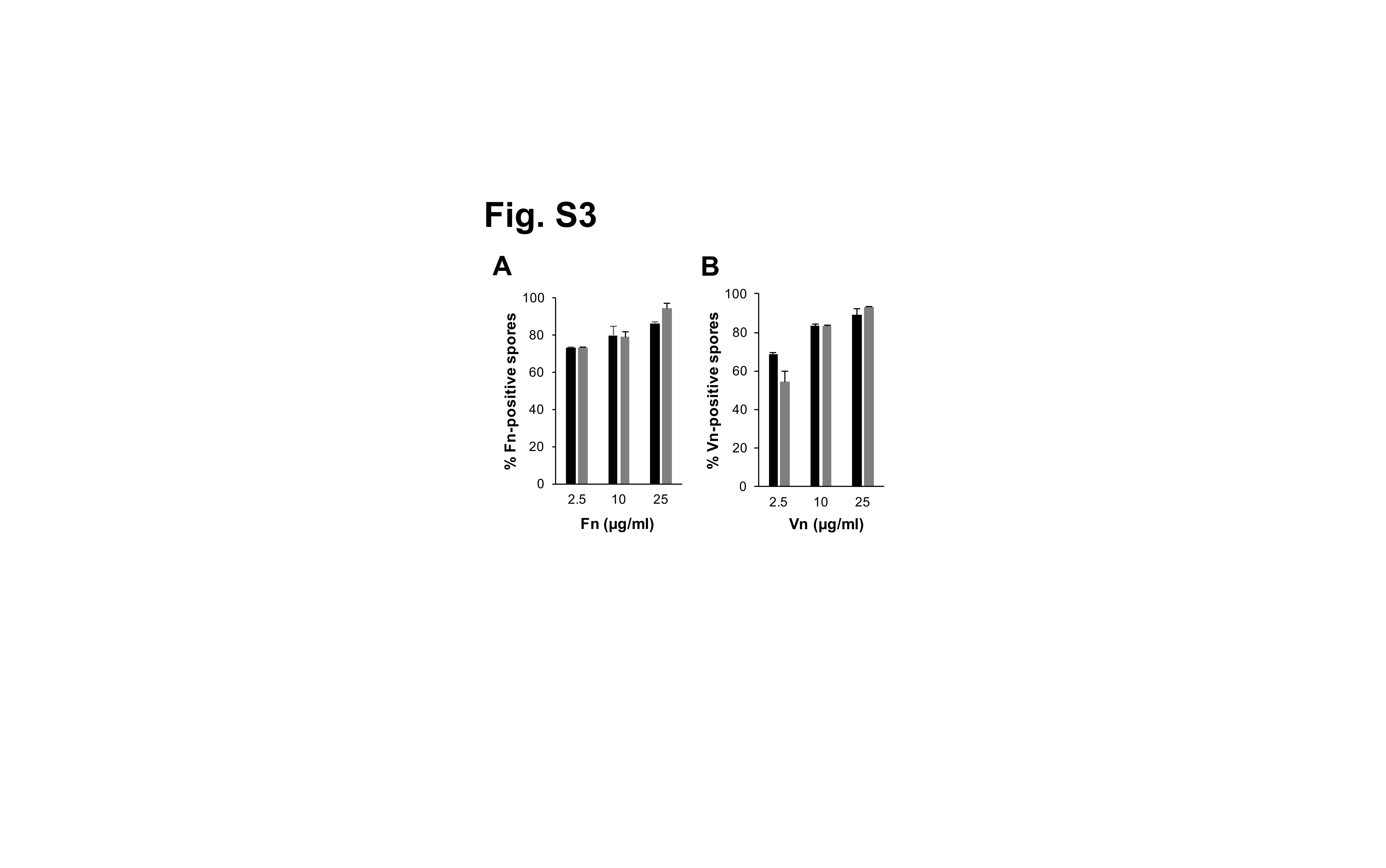

Supplement: Figure S3 — Percentage of fibronectin- and vitronectin-specific immunofluorescent positive spores. C. difficile wild-type (black bars) and cdeC spores were incubated with different concentrations of fibronectin (A) and vitronectin (B) and processed for indirect immunofluorescence as described in the Method section. Immunofluorescence micrographs were analyzed for the presence or absence of visual immunofluorescence signal. A total of 450 spores were counted. Bars represent the mean of three independent experiments and error bars represent standard error from the mean. [file Image3.TIFF]
